# 160. Evaluation of an Intervention to Promote Guideline-Concordant Durations of Antibiotic Therapy in Two Urgent Care Centers

**DOI:** 10.1093/ofid/ofab466.160

**Published:** 2021-12-04

**Authors:** Katherine C Shihadeh, Axel A Vazquez Deida, Cory Hussain, Bryan C Knepper, Lindsey Fish, Michael Breyer, Melody Zwakenberg, Amy Quinones, Bradley Torok, Timothy C Jenkins

**Affiliations:** 1 Denver Health Medical Center, Denver, Colorado; 2 University of Nebraska Medical Center, Tucson, AZ; 3 Denver Health, Denver, Colorado; 4 Denver Health Medical Center, University of Colorado School of Medicine, Denver, Colorado

## Abstract

**Background:**

Antibiotic overuse in urgent cares is common. Despite institutional guidance that recommends ≤ 5 days of therapy for most infections, a prior review found prescribed durations were often longer. This study evaluates the impact of an intervention on guideline-concordant durations of therapy.

**Methods:**

This quasi-experimental study involved two urgent care centers (UC1 and UC2) in an integrated health care system. Prescriptions were included from January 2017 to May 2021 for patients ≥ 18 years of age for one of the following infections identified by ICD10 code: acute bacterial sinusitis, acute otitis media, cellulitis or skin abscess, COPD exacerbation, lower urinary tract infection, or pneumonia. The intervention was implemented in both urgent cares in January 2020 and included sharing baseline duration of therapy data with site directors and staff, providing in-person education on recommended durations of therapy, engaging peer champions, and posting educational flyers. An institutional smart phone application (app) with treatment recommendations for common infections was in place for the entirety of the study. The primary outcome was the proportion of antibiotic durations that were guideline-concordant during the app only and intervention periods in aggregate and by interrupted time-series analysis.

**Results:**

On average, 1583 and 3850 antibiotic prescriptions were prescribed per year in UC1 and UC2, respectively. There was a significant increase in the proportion of guideline-concordant antibiotic prescriptions at the two sites by an absolute value of 20% (p< 0.0001) (Table). By interrupted time-series, the change in slope after the intervention was not statistically significant for UC1 (p= 0.11), UC2 (p= 0.73), or combined (p= 0.61); however, there was a significant increase in prescriptions for ≤ 5 days immediately after the intervention in UC1 (p= < 0.001) (Figure).

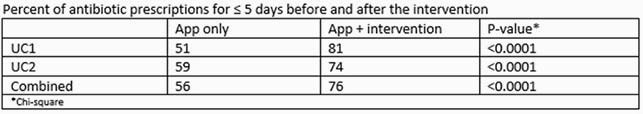

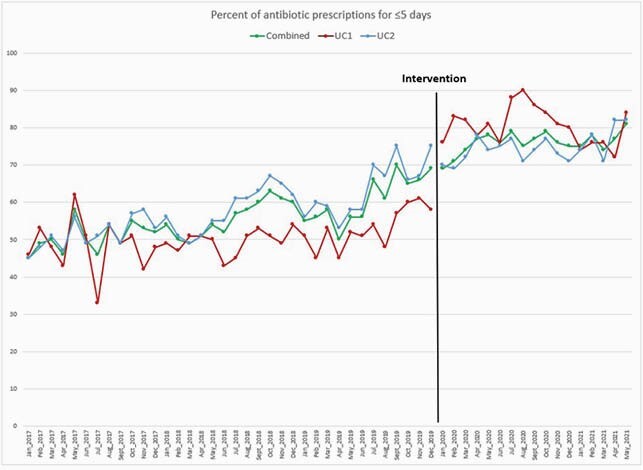

**Conclusion:**

This intervention to promote institutional guideline-concordant durations of therapy resulted in a significant increase in the proportion of antibiotic prescriptions for ≤ 5 days. Preventing prolonged durations of therapy is a potentially effective strategy to reduce antibiotic overuse in urgent cares.

**Disclosures:**

**All Authors**: No reported disclosures

